# Air crescent sign: typical case of invasive pulmonary aspergillosis

**DOI:** 10.11604/pamj.2023.44.130.39058

**Published:** 2023-03-15

**Authors:** Mrinmayee Vijay Mayekar, Neha Phate

**Affiliations:** 1 Department of Respiratory Medicine, Datta Meghe Institute of Higher Education and Research, Wardha, Maharashtra, India

**Keywords:** Invasive aspergillosis, air crescent, post tubercular

## Image in medicine

A 56-year-old male patient presented with complaints of dyspnoea on exercise for the last two months, recurrent hemoptysis for the past two months, and cough with expectoration for the past three months. In addition to this, over the past two months he has been losing weight and his hunger. He had a history of pulmonary Koch´s three years ago, for which he was treated with antitubercular drugs for a period of time equalling six months. In light of the patient's persistent complaints, we decided to perform a chest X-ray on him, followed through a high-resolution computed tomography (HRCT) of his thorax. The outcomes of these investigations hinted to the presence of post-tubercular sequelae in the form of invasive aspergillosis exhibiting an air crescent sign in the patient's left upper lobe. The patient is a well-documented instance of uncontrolled type II diabetes mellitus, which lends credence to our diagnosis.

**Figure 1:**
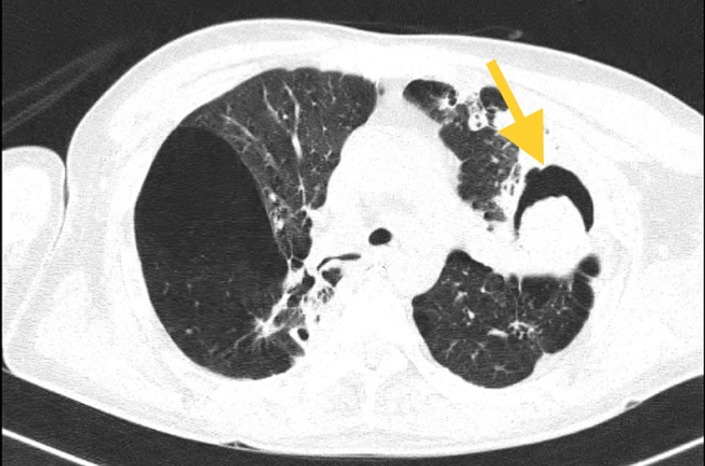
a large thick wall cavitary lesion with air filled surrounding the devitalized parenchyma in left upper lobe with bilateral emphysematous changes

